# Reaching the WHO target of testing persons in jails in prisons will need diverse efforts and resources

**DOI:** 10.1371/journal.pone.0202985

**Published:** 2018-08-30

**Authors:** Sylvie Abel, Lise Cuzin, Séverine Da Cunha, Jean-Marie Bolivard, Laurence Fagour, Charline Miossec, Mathilde Pircher, Marême Thioune, Raymond Césaire, André Cabié

**Affiliations:** 1 Prison Medical Unit, Martinique University Hospital, Fort de France, France; 2 Infectious and Tropical Diseases Unit, Martinique University Hospital, Fort de France, France; 3 INSERM, UMR 1027, Toulouse, France; 4 Virology Laboratory, Martinique University Hospital, Fort de France, France; 5 Parasitology Laboratory, Martinique University Hospital, Fort de France, France; 6 Antilles University, EA4537, Fort de France, France; 7 Inserm CIC1424, Martinique University Hospital, Fort de France, France; University of North Carolina at Chapel Hill, UNITED STATES

## Abstract

**Background:**

The Caribbean is the second most affected region in the world by human immunodeficiency virus (HIV), and HIV prevalence is significantly higher among persons in jails and prisons than in the free population. The aim of our study was to assess the screening rates of HIV, hepatitis B and C, syphilis and human T cell leukaemia virus type 1 among newly-arrived persons in 2014, at Ducos facility in Martinique and the testing process performance.

**Methods:**

This is an observational monocentric study conducted within the prison’s health unit. The study population consisted of all individuals incarcerated between 01/01/14 and 31/12/14. At the initial medical visit, HIV and STI testing were proposed to every newcomer. The rate of acceptance was calculated, as well as the screening process performance.

**Results:**

In 2014 778 new persons were incarcerated, among those, 461 (59.3%) were tested. The main reasons for missing the testing opportunity were due to organization of the judiciary system (persons on electronic monitoring or day parole, transferred or quickly released before completion of the process) or to individual refusal. Finally, 75 persons did not get their results (all of them negative), 41 of them due to the medical staff work overload.

**Conclusions:**

HIV and STI testing rates among newcomers at Ducos have notable room for improvement. The future availability of combined (HIV, HBV, HCV and syphilis) rapid tests may be very useful in case of short term incarceration, if their cost is not prohibitive. Reaching higher levels of testing will also require more resources.

## Introduction

Human immunodeficiency virus (HIV) testing is a crucial part of the combination prevention, is the first step for access and linkage to care, and has been shown to be beneficial both at the individual and at the population level [1, 2]. In France in 2015, HIV testing rate was 81 per 1000 citizens. This proportion was higher in Martinique: 137 per 1000 citizens in the same year. At the national level, 2 tests out of 1000 performed were found to be positive in 2015, 2.3 out of 1000 in Martinique, and even more in the other French Caribbean regions (3.1 in Guadeloupe and 7.4 in French Guyana) [3].

Persons in jails and prisons are considered by the World Health Organization (WHO) as a key population, and at least 90% of them should have access to HIV combination prevention services by 2020 [4]. In France in 2010, 2% of the persons in jails/prisons were found to be HIV positive, by comparison with a global prevalence of 0.23% of the French population [5]. To offer a medical consultation is mandatory during the first days of incarceration of every newcomer. HIV, syphilis, hepatitis B and C testing are to be proposed during this visit, with renewals for those who decline to be tested at the first visit or have risky behaviours while detained [6].

Ducos penitentiary is the only facility in Martinique and comprises jail and prison for adults (both genders) and juveniles. With 750 to 1000 admissions each year, it is permanently overcrowded [7].

The combination of HIV regional epidemiologic data and detention conditions supports active HIV testing in the Ducos facility’s population. The medical staff working in Ducos designed a “screening strategy” including HIV and other sexually transmitted infections testing proposal to every newcomer, all tests being performed on the same venous blood sampling. The complete strategy included opt-in proposal, actual blood sampling, results communication to the person, and linkage to care if necessary. This strategy was routinely executed in Ducos for many years, without evaluation. The aim of our study was to assess the efficiency of the strategy and to look for ways of improvement if needed.

## Methods

This monocentric observational study was conducted in the medical unit of the Ducos facility (Martinique, FWI). The administrative and medical charts of all subjects imprisoned between January the 1^st^ and December the 31^st^ 2014 were analysed. From the administrative charts, we extracted the date of incarceration and the mode of detention (accused or sentenced, either in jail, day parole, or assigned to wear an ankle monitoring device after a few days in the facility). If the person was transferred or returned to freedom during the study period, the date of release was noted. The unit collects medical information, with the subjects’ written consent, via the Nadis^®^ electronic medical record [8]. For about 20 years, HIV, hepatitis B virus (HBV) or hepatitis C virus (HCV), human T cell leukaemia virus type 1 (HTLV-1) and syphilis testing are to be proposed to all subjects during the first medical visit, unless tests performed recently in another facility are available. From the Nadis^®^ database we extracted information collected during the first medical visit: date of visit, age, gender, place of birth, and drugs consumption habits. According to French law, these data collected for medical care can be used for research purposes. Data from the administrative and medical files were extracted mid-2015 by the facility medical unit staff and a de-identified data set was provided to the research staff for analysis. The tests proposal, acceptance or refusal, and the actual date and results of testing were collected. HIV infection, presence of HBs antigen, anti-HBs and anti-HBc antibodies, and HTLV-1 were coded as positive or negative. Syphilis was defined as positive if the Treponema Pallidum Hemagglutinations Assay was positive and Venereal Disease Research Laboratory titre was 4 or more. Dates of results announcement to the person and linkage to care in case of a new diagnosis were recorded.

In case of the absence of first medical visit during the first week of incarceration, the reason was noted: released in the first days, transfer, subject on day parole or assigned to wear an ankle monitoring device (thus not present in the facility long enough), subject refusal, or appointment not planned by the staff.

In case of the absence of tests proposal during the visit, the reasons were also noted: previous test in a recent incarceration, or test already known to be positive. In case of tests refusal by the person, another visit was to be proposed, and the date of this visit was collected. If testing was performed later-on while the subject was still in the facility, the date of testing was collected.

Continuous variables are described by medians and 25% interquartile (Q1-Q3). Categorical variables are described by proportions. Analysis was performed by use of Stata 12 (StataCorp, College Station, USA).

## Results

**Fig 1:**
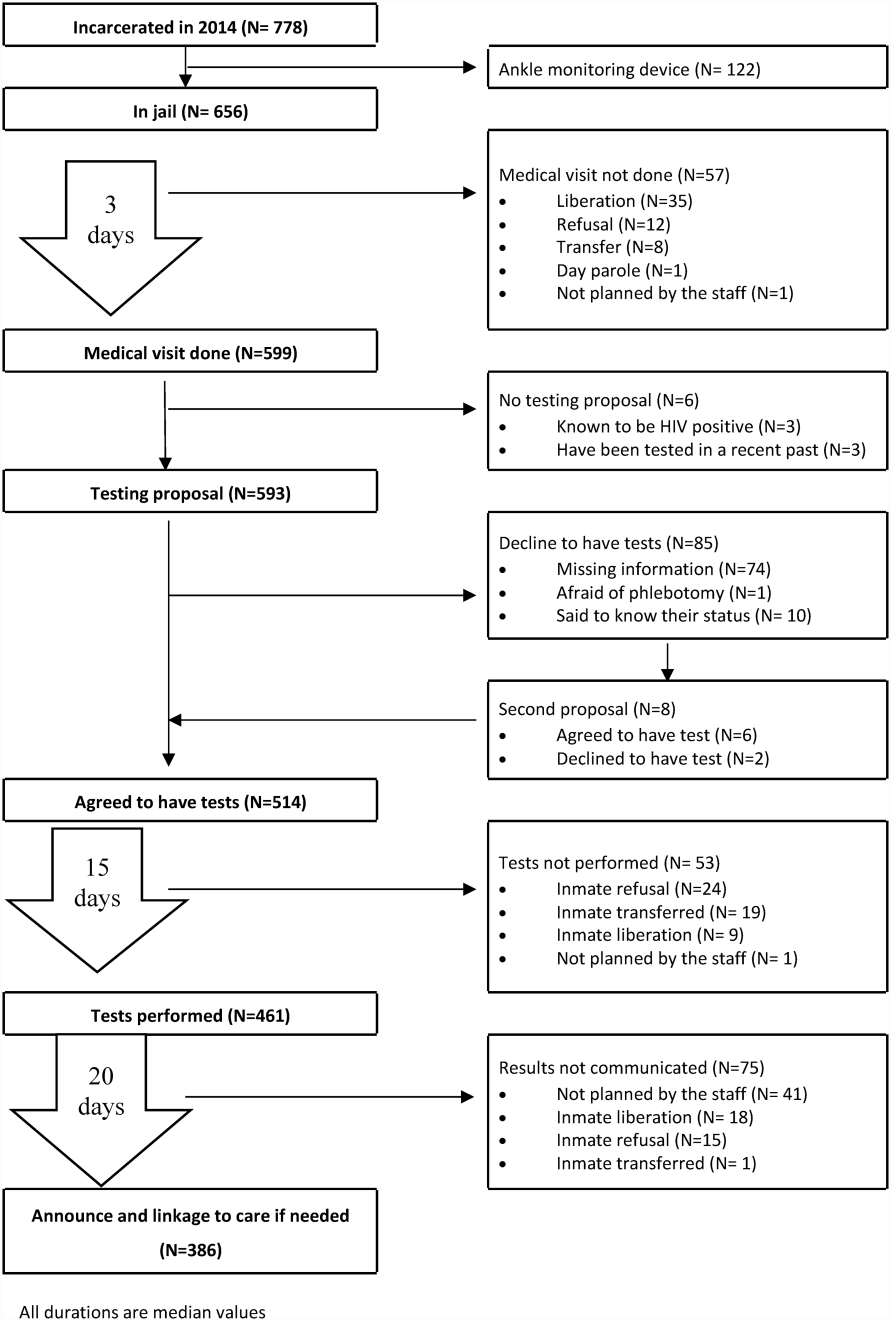
Flow chart of persons incarcerated in 2014, medical visits, STI testing proposal, testing agreement, actual testing, and announce with linkage to care if needed, in the Ducos Facility (Martinique, FWI).

During the study period, 778 persons have been imprisoned at Ducos facility, 327 of them being accused (42%) and 451 sentenced. The majority of them were men (95.5%), young (median age 29 years, IQR 23–38), and 28.8% were active drug users. Among them, 122 (15.7%) were assigned to wear an ankle monitoring device and thus because they did not stay long enough in the facility the medical visit was not feasible. Fig 1 shows the flow chart of the subjects along the process.

Early liberation (35 persons) or transfer (eight persons) did not allow the staff to plan a medical visit. Moreover, one was on day parole, thus not present at the facility during the medical unit working hours. Finally, 12 declined to meet the medical staff, and one was not planned by the staff. Thus 599 persons (77% of the newcomers) met the medical staff in the first days of incarceration (median 3 days, IQR 2–4), and 593 (76.2%) were offered the tests (in three cases the person was known to have been tested in current incarceration before being transferred to Ducos, and in three cases the doctor did not propose the tests).

Tests were accepted by 514 persons (66.1% of the total population, 85.8% of those who came to the visit, 86.7% of those who were proposed), in some cases after a second encounter with the medical staff. Unfortunately, for 74 persons refusing to be tested the reason of refusal was not written in the medical chart. Testing was actually performed for 461 persons (59.2% of the total population) after a median time of 15 days after the medical visit (IQR 1–18). The reasons for not performing the tests were refusal (24 persons), transfer (19 persons), liberation (nine persons), and one was forgotten by the staff.

Tests results were announced during a subsequent medical visit, in a median time of 20 days (IQR 13–34) after testing, to 386 persons (49.6% of the total population, 83.7% of the tested population). Reasons for missed announcements were liberation (18 persons), transfer (one person), refusal to come to the visit (15 persons), or the visits were not planned (41 persons).

**Fig 2:**
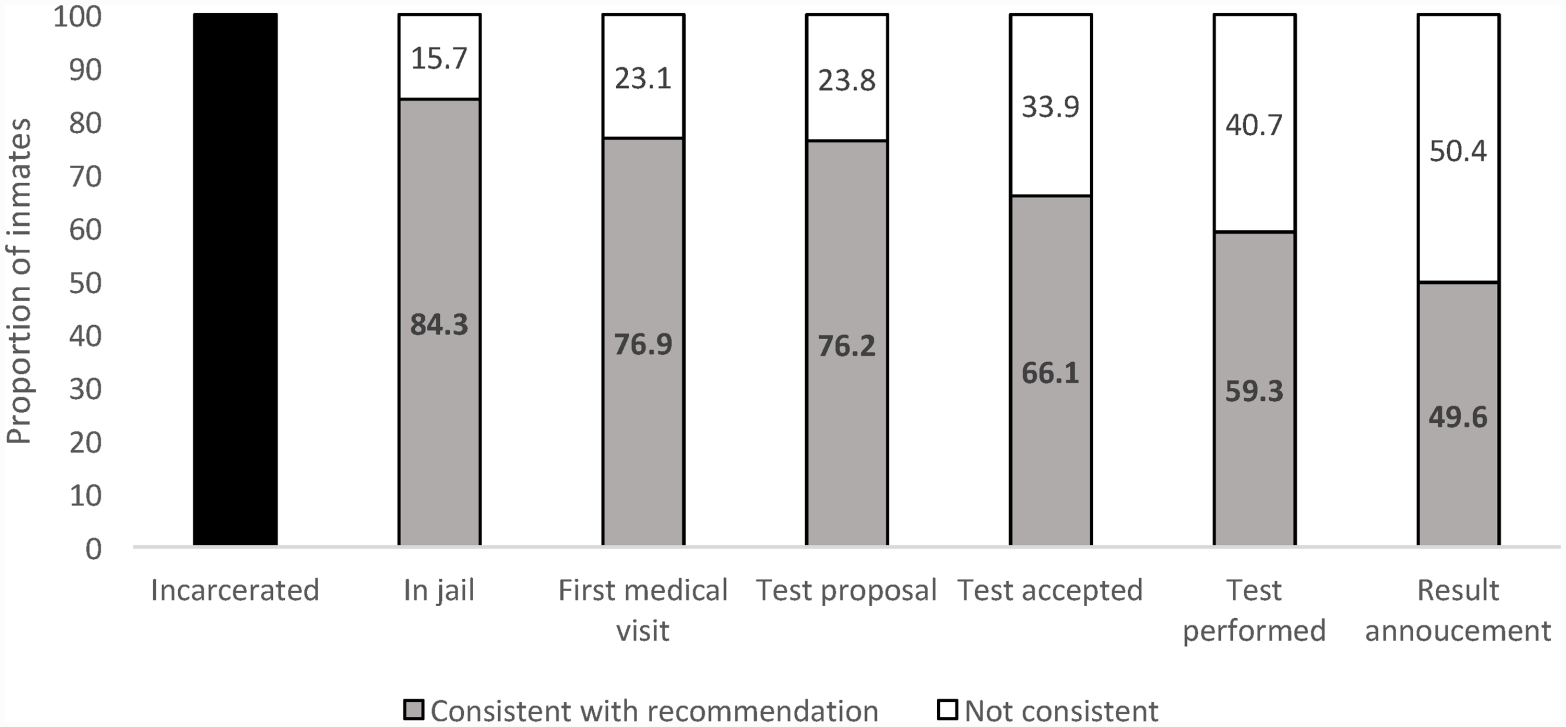
Cascade of sexually transmitted infections testing in the Ducos facility, Martinique (FWI) in 2014.

Finally, 461 persons were tested (59.3% of the total population) in a median of 20 days (IQR 15–23) after incarceration and 386 (49.6% of the total population) have been informed of their status regarding HIV, HBV, HCV, HTLV1 and syphilis in a median time of 40 days after incarceration (IQR 31–56). If we consider only the persons actually staying at Ducos on days and nights, the proportion of prisoners being tested was 70.3% and 58.9% had both the tests and information on the results. This care cascade is synthetized in Fig 2.

HIV was found to be positive in four persons, all being already aware. Median time between HIV testing and result notification was 9.5 days (IQR 4.5–14). Two persons received potent anti-retroviral therapy while incarcerated, the other two were transferred.

Chronic hepatitis B virus infection (presence of HBs antigen) was found in four persons, three of them being already aware. Among the four cases of active HBV, one received treatment, two were monitored but not treated, and one was transferred. Anti-HBs antibodies were present for 237 persons (51.4% of the tested population) and anti-HBc in 25 (5.4%). Median time between HBV testing and result notification was 33 days (IQR 14–47). Among the 220 persons non-immune to HBV, 130 received a first vaccination.

All HCV tests were negative. HTLV-1 was positive in 1 case. Syphilis was diagnosed in eight cases (1.7% of tested persons). Median time between syphilis testing and result notification was 15.5 days (IQR 12.5–18.5). All syphilis cases received adequate dosages of benzathin-penicillin and serological follow-up.

## Discussion

Despite a clear involvement of the medical staff working in Ducos, the only jail/prison in Martinique, rates of testing for HIV were below the WHO target. This is not different from what was reported by Rumble et al. in a systematic review. They found that opt-in HIV testing programs resulted in 25 to 72% of persons in jails/prisons being tested [9]. Many steps in the cascade showed potential for improvement. First of all, 21.3% of the newcomers were not staying inside the prison for a sufficient time to attend to the medical visit. The transient nature of this justice population may require new strategies to improve linkage to care [10]. The potential use of rapid tests in settings with fast turnaround [11], followed by immediate linkage to care, could lessen the part of missed testing opportunities. The future availability of combined (HIV, HBV, HCV and syphilis) rapid tests may be very useful in jail settings, if their cost is not prohibitive. Nevertheless, restricting the analysis to the persons who actually stayed in the facility, 17.3% declined to be tested, even after a second proposal during incarceration. The proportion of persons declining to be tested in our study is much lower than in other studies in the Caribbean region [12], and is in line with other studies [9]. The confidential proposal during one-to-one interviews between the prisoner and the doctor may have helped to reduce the refusal rate. As in any settings proposing testing on an opt-in basis, we expected some refusals. The fact that acceptance was not 100% underlines the persons’ ability to refuse to be tested. Other studies failed to link HIV stigma to test acceptance, but showed a relation between test acceptance and HIV knowledge [13]. Thus enhancing the knowledge on HIV risk factors, treatment and prognosis may help to enlarge the proportion of persons willing to be tested [14]. In French facilities, health care is entrusted to the nearest public hospital. This separation between health and penitentiary systems aims to protect confidentiality as much as possible. This should also be emphasized to newcomers while testing is proposed, as to decrease the rate of refusals due to fear of confidentiality breakage. Finally, only 8 persons out of 85 who declined to be tested at entry had a second proposal. This could be explained by permanent work overload and overcrowding. Closing this part of the gap may require more health manpower in Ducos.

The median delay of 15 days between test proposal by the doctor and actual phlebotomy by the nurse is in part responsible for 3.9% of persons agreeing but not actually tested, as they were transferred or returned to freedom during this time lapse. Moreover, 4.7% of the agreeing population had time to change their mind during this time lapse. Among those who were finally tested, 4.1% were transferred or returned to freedom without knowledge of the tests results. Fifteen prisoners (20% of the persons without communication of results) refused to come to an announcement visit. Importantly all those with a positive test for one or several of the tested diseases were informed and linked to care. Of great importance are the 41 persons who could not be informed of their results because de staff could not plan a medical visit, due to chronic work overload. Here again, the use of rapid tests with immediate results communication could be helpful [15], keeping the available manpower for testing prisoners at high risk of acute infection.

Observed prevalence of HIV was 0.9%, less than the prevalence found in the French PREVACAR study [5]. No HCV infection was detected during this study. Those facts could be related with the absence of drug injection practices in Martinique, most of the persons considered as drug users being crack smokers. HTLV-1 was found in only 1 case out of 461 (0.2%). HTLV-1 prevalence is not well known in Martinique, one study more than 15 years ago estimated that it was 1.93%, in pregnant women [16]. Regarding HBV, it is important to note that 48.6% of the tested persons had an indication of hepatitis B vaccination (absence of detectable anti-HBs antibodies). Half of them actually initiated an immunization process.

The main limit of our study is the retrospective analysis of data collected during the first visit. This results on missing information on the reason for declining test in 74 out of 79 refusing persons.

## Conclusions

HIV and STI testing rates among newcomers in the Martinique facility have notable room for improvement. Confidentiality may be a concern for persons who are therefore reluctant to be tested. There is a need to raise awareness as to increase testing acceptance. Reaching higher levels of testing will also require more resources, and rapid tests could be useful in jails.

## Supporting information

S1 Dataset (ZIP)
